# Evaluating Modeling and Validation Strategies for Tooth Loss

**DOI:** 10.1177/0022034519864889

**Published:** 2019-07-30

**Authors:** J. Krois, C. Graetz, B. Holtfreter, P. Brinkmann, T. Kocher, F. Schwendicke

**Affiliations:** 1 Department of Operative and Preventive Dentistry, Charité–Universitätsmedizin Berlin, Berlin, Germany; 2 Clinic of Conservative Dentistry and Periodontology, University of Kiel, Kiel, Germany; 3 Department of Restorative Dentistry, Periodontology, Endodontology, Preventive Dentistry and Pedodontics, Dental School, University Medicine Greifswald, Greifswald, Germany

**Keywords:** periodontitis, treatment planning, regression analysis, biostatistics, dental, periodontal disease

## Abstract

Prediction models learn patterns from available data (training) and are then validated on new data (testing). Prediction modeling is increasingly common in dental research. We aimed to evaluate how different model development and validation steps affect the predictive performance of tooth loss prediction models of patients with periodontitis. Two independent cohorts (627 patients, 11,651 teeth) were followed over a mean ± SD 18.2 ± 5.6 y (Kiel cohort) and 6.6 ± 2.9 y (Greifswald cohort). Tooth loss and 10 patient- and tooth-level predictors were recorded. The impact of different model development and validation steps was evaluated: 1) model complexity (logistic regression, recursive partitioning, random forest, extreme gradient boosting), 2) sample size (full data set or 10%, 25%, or 75% of cases dropped at random), 3) prediction periods (maximum 10, 15, or 20 y or uncensored), and 4) validation schemes (internal or external by centers/time). Tooth loss was generally a rare event (880 teeth were lost). All models showed limited sensitivity but high specificity. Patients’ age and tooth loss at baseline as well as probing pocket depths showed high variable importance. More complex models (random forest, extreme gradient boosting) had no consistent advantages over simpler ones (logistic regression, recursive partitioning). Internal validation (in sample) overestimated the predictive power (area under the curve up to 0.90), while external validation (out of sample) found lower areas under the curve (range 0.62 to 0.82). Reducing the sample size decreased the predictive power, particularly for more complex models. Censoring the prediction period had only limited impact. When the model was trained in one period and tested in another, model outcomes were similar to the base case, indicating temporal validation as a valid option. No model showed higher accuracy than the no-information rate. In conclusion, none of the developed models would be useful in a clinical setting, despite high accuracy. During modeling, rigorous development and external validation should be applied and reported accordingly.

## Introduction

Personalized medicine relies on prediction models, which quantify a patient’s or a tooth’s risks for experiencing a disease (periodontitis) or a complication (tooth loss) in the future. To do so, prediction models learn patterns from available data (training) and are then applied to other data (testing/prediction). Calls for more rigorous development and validation of such models have been raised (Kundu et al. 2017).

A range of factors should be considered by modelers during the development and validation of prediction models.

1) Model complexity. For example, regression models or decision trees result in interpretable models that are easier to communicate to decision makers. They usually also consider a predetermined, smaller number of prediction variables. Alternative, more complex ensemble models, such as gradient boosting models, could be applied but are more difficult to interpret by a human decision maker and may require significant computational resources. They also allow one to consider a larger number of prediction variables, while model applicants may want to gauge the model by assessing if “logical” predictors (e.g., those known to be associated with the disease or event) are employed by the model for prediction making.

2) Sample size and imbalanced class sizes. In general, most classification algorithms perform better with larger sample sizes and more balanced class sizes. For imbalanced data sets (e.g., most tooth loss data sets, where tooth loss occurs less often than tooth retention), specific model performance metrics are more appropriate than others.

3) The prediction period—that is, if predictions are made over short-, medium-, or long-term periods.

4) The training and testing (i.e., validation) strategy. Prediction models may not be externally validated but trained and tested in the same cohort (internal validation)—for example, by applying a training-test split, where a portion of the data set is assigned as a training set and the other part as a holdout test set (“out-of-sample performance”). In dentistry, in many cases, not even such internal validation is performed, but only the in-sample performance is reported (training and testing on the same data, where not even a separate test data set is used; Mejare et al. 2013; Du et al. 2018; Schwendicke et al. 2018). If a prediction model, however, will be applied in clinical reality, the “test” data set will always stem from another cohort. Hence, external validation—for example, testing on a sample from a different period or a different population—is usually recommended over internal testing to gauge a model’s generalizability (Zhan et al. 2014; Steyerberg and Harrell 2016; Steyerberg et al. 2017). Only generalizable models provide additional value to the treating dentist.

Predicting tooth loss in patients with periodontitis is relevant for treatment planning and decision making before and during active and supportive periodontal therapy (APT and SPT). Predicting during treatment planning if teeth can or cannot be retained may lead to less invasive treatment plans, better health outcomes, and reduced long-term costs (Schwendicke et al. 2017; Schwendicke et al. 2018). It is unclear, at present, what relevance the different outlined steps for developing and validating tooth loss predictions models have on their apparent predictive power (in sample) and true predictive power (out of sample, ideally externally validated). We aimed to evaluate the impact of model complexity, sample size, prediction periods and training, and validation strategies on tooth loss prediction models in patients with periodontitis.

## Methods

This study follows the TRIPOD reporting guidelines (Moons et al. 2015) for developing prediction models and the reporting guidelines to address common sources of bias in risk model development (RiGoR; Kerr et al. 2015). Note that our aim was not to develop a model for clinical application at this stage but to demonstrate the impact of different development and validation steps on model performance. Our analyses hence serve to assess how vulnerable prediction performance is to different aspects of modeling (e.g., building the model on too small sample sizes, training and validating it only internally).

### Source of Data, Participants, and Therapy

Two cohorts, 1 at the University of Kiel and 1 at the University of Greifswald, were established in the 1980s and 1990s, respectively. Patients were consecutively included. During APT (T0 to T1), patients received mainly nonsurgical mechanical root debridement, with or without surgical therapy. Patients received SPT (T1 to T2) for ≥9 y (Kiel) and ≥4 y (Greifswald; T1 until last visit, T2) at individualized intervals. The study was approved by the local ethics committees (Kiel: D489/13; Greifswald: BB91/10). Details on the cohorts and treatment concepts are available in the Appendix.

### Outcome

The prediction outcome was tooth loss during SPT (T1 to T2). Tooth loss was not restricted to that due to periodontitis, as in many cases 1) the reasons for tooth removal were multiple or 2) the reasons could not be ascertained. Also note that we did not include tooth loss during APT (T0 to T1) in our assessment, as this is mainly guided by therapy decisions and thus not the subject of prediction making. Tooth loss was not assessed blind; that is, dentists were likely aware of the initial dental status. The risk of bias stemming from this, however, is very low, as tooth loss is a hard outcome and not easy to bias.

### Predictors

From both cohorts, 4 patient-level and 6 tooth-level predictors were available. Predictors were not assessed blindly but within routine care. As all predictors were recorded prior to the outcome (tooth loss) being able to occur, the risk of bias stemming from this is very low. Note that some predictors were assessed only at T0, not T1, usually to avoid repeated radiographic assessment. We do not assume any significant change in the status to have occurred from T0/T1 onward. On the patient level, we assessed the following: age and sex at T0; the number of teeth after APT (i.e., at T1); and smoking status, which was assessed categorically as never/former (i.e., quit >5 y ago) and current (Lang and Tonetti 2003) at T0. The number of smoked cigarettes was not assessed for all patients and hence could not be employed. Also note that smoking status may have changed over the long-term follow-up, which we did not account for, as prediction making is mainly interested in the initial smoking status to allow better decisions early on.

Diabetes status was not uniformly assessed and comprehensively available in both cohorts and is thus not included here.

On the tooth level, a full dental status was recorded once yearly. Data for third molars were not included. The following variables were available:

- Tooth type (molar or not)
- Probing pocket depths (PPDs), which were evaluated at 6 sites per tooth; only the maximum recorded PPD per tooth at T1 was used.
- Mobility at T1, classified by degrees 0 to 3 (Lindhe and Nyman 1977)
- Relative radiographic bone loss at T0, categorized into ≤25%, >25% to 70%, and >70% (Graetz et al. 2011)
- The furcation involvement of molars according to Hamp et al. (1975) at T1; only the highest degree of furcation involvement for each molar was used (McGuire and Nunn 1996).
- Dental arch (lower or upper)

Note that variations in periodontal therapy (e.g., tooth splinting, root resection, and tunneling, as described in the Appendix) were not employed as predictors, as these may reflect dentists’ preference and expertise as much as periodontal affection. Also, further treatments provided during SPT or SPT intervals were not accounted for, as these will not be known at baseline, when predictions are made. Notably, though, they likely have an impact on tooth loss.

### Statistical Experiments

A number of experiments were performed to assess the impact of model complexity, sample size, prediction periods, and training and validation strategies on the predictive power of a model.

1) Complexity: We considered 4 binary classification models. Sorted by complexity (defined by the number of tuneable hyperparameters), these were logistic regression (logR), recursive partitioning (RPA), random forest (RFO), and extreme gradient boosting (XGB). We assessed the predictive performance of these models and further explored which predictors were preferably employed by different models. Further details are provided in the Appendix.

2) Sample size: To evaluate the impact of the sample size, we dropped 10%, 25%, and 75% of individuals at random.

3) Prediction period: The impact of prediction periods was evaluated by censoring the follow-up period to 10, 15, and 20 y or not at all. Note that this analysis was performed only for the Kiel cohort, as this cohort showed sufficiently long follow-up. If a tooth was lost after the censoring period, this outcome was not included for modeling.

4) Training and validation strategies: Each model was trained on a training set and evaluated via resampling and cross-validation (thereby providing a confidence interval) and, apart from that, on a holdout test set. All applied data set splits were stratified with respect to the outcome variable to keep the patient-level tooth loss in the training and test set as close as possible to that in the full data set. To evaluate different model validation schemes, all 4 binary classification models (RPA, RFO, XGB, logR) were applied on 6 validation scenarios, representing different training and test approaches. For scenario S1, the baseline scenario, we applied a stratified random 75/25 split on the full data set (75% training of the combined Kiel-Greifswald cohort, 25% test data also in the complete cohort). For scenarios S2 and S3, we split the data along the centers of data collection (training in Kiel and test in Greifswald and vice versa). For scenarios S4 and S5, we split the data within each center of data collection (training in Kiel and test in Kiel, training in Greifswald and test in Greifswald). In S6, we evaluated the impact of temporally external validation by splitting the data set into a training set (constituting the cohort from 1980 to 1995) and a test set (constituting the cohort from 1995 onward). Note that for S6, only Kiel data were used, as sufficiently long follow-up periods were available.

Note that data were analyzed at the tooth level for all models. In a sensitivity analysis (see Appendix) we applied logR with a random subject term being introduced (mixed effect logR) to account for clustering effects. This, however, did not affect the model performance (Appendix Tables 1 and 2). While implementation of the clustered structure in the other models was not feasible, thus prohibiting us from exploring how the within-mouth correlation of teeth affects their accuracy and confidence intervals, we assumed the impact of clustering to be limited.

### Modeling and Performance Metrics

We used the area under the receiver operating characteristic curve, the accuracy and its 95% CI, as well as sensitivity and specificity as performance metrics. A class threshold of 0.5 was applied. Furthermore, we reported the no-information rate (NIR), which corresponds to the percentage of the majority class in the training data. The NIR provides information on how accurate guessing would be if a “prediction” would be built on only “predicting” this majority class. In case the events are very rare, for example, guessing that no event occurs can have a high accuracy. A 1-sided binomial hypothesis test was applied to test if the predicted accuracy was higher than the NIR. Further details are provided in the Appendix.

## Results

### Sample and Tooth Loss

The characteristics of both cohorts are shown in Table 1. The Kiel cohort was larger and SPT longer than in Greifswald. The age at T0 was not significantly different between the cohorts, while in Greifswald, the mean tooth loss per patient was higher than in Kiel. Patients who lost teeth were initially older and more often smokers than nonsmokers or former smokers. On the tooth level, molars; teeth with higher PPD, furcation involvement, and bone loss; mobile teeth; and lower teeth were more often lost (Table 2).

**Table 1.** Characteristics of the Sample at Different Time Points.

	*n* or Mean ± SD	
Parameter	Kiel	Greifswald
Patients, male:female	164:226	102:135
Age at T0, y	45.9 ± 10.2	47.1 ± 10.4
SPT (T1 to T2), y	18.2 ± 5.6	6.6 ± 2.9
Smoker:former smoker:never smoker (T0)	50:88:252	31:81:125
No. of tooth loss / (patient × year) (SPT)	0.11 ± 0.15	0.14 ± 0.58

APT ranged from T0 to T1 (first visit to last APT visit) and SPT from T1 to T2 (last APT visit to last SPT visit).

APT, active periodontal therapy; SPT, supportive periodontal therapy.

**Table 2.** Distribution of Tooth Loss according to Different Patient- and Tooth-Level Variables in the Full (Unrestricted) Data Set.

Patient Level	Patients with Tooth Loss, *n* (%)	Tooth Level	Teeth Lost, *n* (%)
Age at T1, y ^a^		Tooth type	
Lost	47.1 ± 9.5	Molar	466 of 3,107 (15.0)
Retained	45.6 ± 10.9	Nonmolar	414 of 8,544 (4.8)
Smoking status		Probing pocket depth, mm	
Never	191 of 377 (50.7)	<5	529 of 9,848 (5.4)
Former smoker	75 of 169 (44.4)	5 to 7	294 of 1,654 (17.8)
Current smoker	50 of 81 (61.7)	>7	57 of 149 (38.3)
Sex		Furcation involvement	
Male	132 of 266 (49.6)	Grade 0 to 1	682 of 10,813 (6.3)
Female	184 of 361 (51.0)	Grade 2 to 3	198 of 838 (23.6)
		Bone loss	
		≤25	104 of 3,810 (2.7)
		>25 to 50	311 of 5,253 (5.9)
		50 to 70	325 of 2,153 (15.1)
		>70	140 of 435 (32.2)
		Mobility	
		0	685 of 10,685 (6.4)
		1	115 of 645 (17.8)
		2	48 of 257 (18.7)
		3	32 of 64 (50.0)
		Dental arch	
		Lower	530 of 5,293 (10.0)
		Upper	350 of 6,358 (5.5)

*N* = 627 patients, 11,651 teeth.

a Mean ± SD.

### Impact of Model Complexity

**Figure 1.**
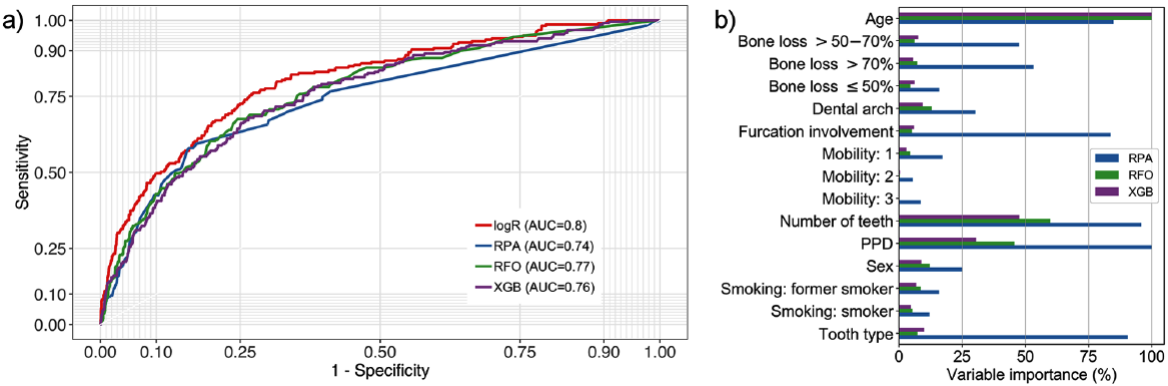
Baseline models. (**a**) Receiver operating characteristic curves of the different models and AUC values. The different models showed similar performance. (**b**) Standardized variable importance for different models (see Appendix for details). Different models built on different predictor variables. AUC, area under the curve; logR, logistic regression; PPD, probing pocket depth; RFO, random forest; RPA, recursive partitioning; XGB, extreme gradient boosting.

Results from different models trained and tested in the complete 2-cohort data set (base case, scenario S1) are shown in Table 3; the receiver operating characteristic curve is shown in Figure 1a. All models showed low sensitivity and high specificity. The classification accuracy was generally high (95% CI, 0.90 to 0.95). Notably, the NIR was similarly high, and none of the models performed significantly better than making informed guesses based on prior knowledge of the class proportions.

**Table 3.** Metrics for the Different Model Validation Schemas.

	AUC (95% CI)						
Model	Test	Training	Specificity	Sensitivity	Accuracy (95% CI)	NIR	*P* Value
Scenario 1: Base case. Training set: 8,821 teeth, 472 patients. Test set: 2,830 teeth, 155 patients.							
RPA	0.74	0.76 (0.74 to 0.78)	0.97	0.13	0.91 (0.9 to 0.92)	0.92	0.999
RFO	0.77	0.84 (0.83 to 0.85)	0.99	0.1	0.92 (0.91 to 0.93)	0.92	0.846
XGB	0.76	0.84 (0.84 to 0.85)	0.98	0.16	0.91 (0.9 to 0.92)	0.92	0.962
logR	0.8	0.8 (0.79 to 0.81)	0.99	0.1	0.92 (0.91 to 0.93)	0.92	0.406
Scenario 2: Training Greifswald–test Kiel. Training set: 4,141 teeth, 237 patients. Test set: 7,510 teeth, 390 patients							
RPA	0.62	0.72 (0.68 to 0.77)	1.0	0.03	0.9 (0.9 to 0.91)	0.9	0.402
RFO	0.75	0.9 (0.88 to 0.91)	1.0	0.0	0.9 (0.9 to 0.91)	0.9	0.587
XGB	0.72	0.89 (0.88 to 0.9)	1.0	0.03	0.9 (0.9 to 0.91)	0.9	0.632
logR	0.77	0.84 (0.82 to 0.86)	1.0	0.03	0.9 (0.9 to 0.91)	0.9	0.343
Scenario 3: Training Kiel–test Greifswald. Training set: 7,510 teeth, 390 patients. Test set: 4,141 teeth, 237 patients							
RPA	0.76	0.75 (0.73 to 0.77)	0.95	0.21	0.93 (0.92 to 0.93)	0.96	≥0.999
RFO	0.78	0.84 (0.83 to 0.85)	0.97	0.19	0.94 (0.93 to 0.94)	0.96	≥0.999
XGB	0.79	0.83 (0.83 to 0.84)	0.98	0.2	0.95 (0.94 to 0.96)	0.96	≥0.999
logR	0.82	0.8 (0.79 to 0.8)	0.98	0.21	0.96 (0.95 to 0.96)	0.96	0.989
Scenario 4: Training Kiel–test Kiel. Training set: 5,694 teeth, 294 patients. Test set: 1,816 teeth, 96 patients							
RPA	0.74	0.73 (0.71 to 0.75)	0.95	0.15	0.88 (0.86 to 0.89)	0.91	≥0.999
RFO	0.77	0.84 (0.83 to 0.84)	0.98	0.12	0.9 (0.89 to 0.92)	0.91	0.73
XGB	0.73	0.84 (0.83 to 0.85)	0.97	0.17	0.9 (0.88 to 0.91)	0.91	0.949
logR	0.81	0.79 (0.78 to 0.8)	0.99	0.15	0.91 (0.89 to 0.92)	0.91	0.488
Scenario 5: Training Greifswald–test Greifswald. Training set: 3,127 teeth, 178 patients. Test set: 1,014 teeth, 59 patients							
RPA	0.68	0.66 (0.6 to 0.71)	1.0	0.04	0.95 (0.94 to 0.97)	0.95	0.48
RFO	0.77	0.88 (0.86 to 0.9)	1.0	0.08	0.95 (0.94 to 0.97)	0.95	0.422
XGB	0.76	0.88 (0.86 to 0.9)	1.0	0.08	0.95 (0.94 to 0.96)	0.95	0.538
logR	0.75	0.85 (0.83 to 0.86)	1.0	0.02	0.95 (0.94 to 0.96)	0.95	0.594
Scenario 6: Training Kiel–test Kiel. Training set (cohort from 1980 on, censored by 15 y) 4,397 teeth, 233 patients. Test set (cohort from 1995 on, censored by 15 y): 3,113 teeth, 157 patients							
RPA	0.72	0.75 (0.73 to 0.78)	0.97	0.15	0.9 (0.89 to 0.91)	0.91	0.999
RFO	0.77	0.84 (0.82 to 0.86)	0.99	0.1	0.91 (0.9 to 0.92)	0.91	0.838
XGB	0.75	0.85 (0.83 to 0.86)	0.98	0.14	0.91 (0.89 to 0.92)	0.91	0.939
logR	0.81	0.8 (0.78 to 0.82)	0.99	0.11	0.91 (0.9 to 0.92)	0.91	0.39

Six scenarios were tested, with 4 models being tested in each scenario. AUC, sensitivity, specificity, accuracy, NIR, and *P* value for the comparison between the accuracy and NIR for the different models.

AUC, area under the curve; logR, logistic regression; NIR, no-information rate; RFO, random forest; RPA, recursive partitioning; XGB, extreme gradient boosting.

The area under the curve (AUC) of each model on the training data set (in-sample AUC) was consistently higher than the AUC on the test data set (out-of-sample AUC). Especially more advanced models were prone to overfitting: they nearly perfectly “learned” the available data of the training data set, and when applied to the test data set, the AUC dropped. More complex models did not have significant advantages over simpler models. The different models also relied on different predictor variables, as shown in Figure 1b. RPA used age, number of teeth at T1, PPD, furcation involvement, and tooth type very often, while other models mostly built on age, PPD, and number of teeth at T1.

### Impact of Sample Size and Prediction Period

**Figure 2.**
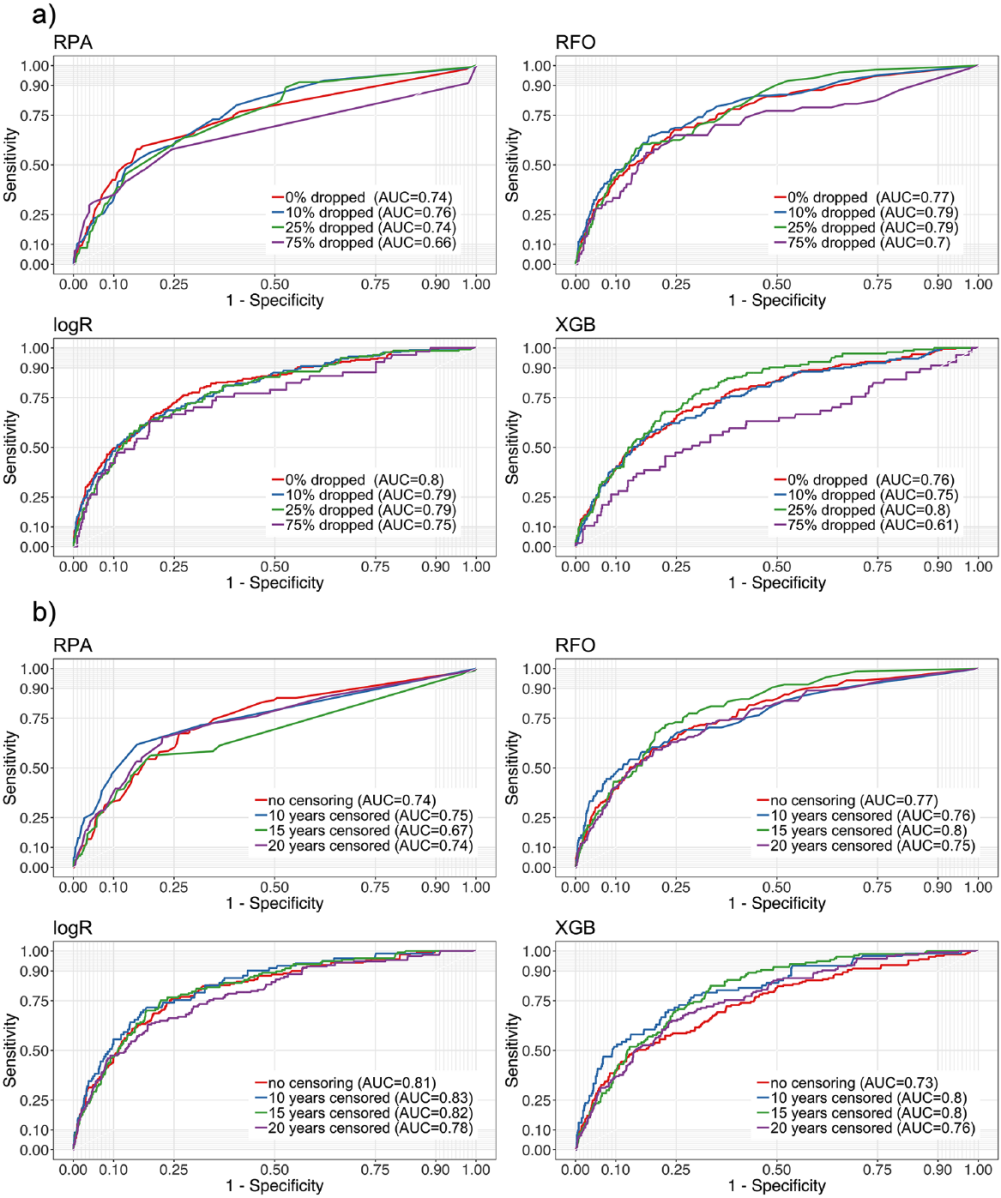
Analyses on restricted data sets, testing the impact of (**a**) sample size and (**b**) prediction periods. Receiver operating characteristic curves of the different models and AUC values are displayed. (a) Dropping individuals from the cohort was performed to shrink it, assessing the impact of cohort size on model performance. Lower sample sizes came with lower model performance. (b) Censoring the prediction period was performed to assess if short-term predictions are more accurate to make than long-term ones. Prediction periods had only limited impact on model performance. AUC, area under the curve; logR, logistic regression; RFO, random forest; RPA, recursive partitioning; XGB, extreme gradient boosting.

We further performed analyses on restricted data sets to explore the impact of sample size and prediction period (Fig. 2). Randomly dropping individuals from the cohort was performed to shrink it and assess the impact of sample size (Fig. 2a). All models tended to lose accuracy when trained on smaller data sets; the degree of loss was similar across models. However, for more complex models, such as XGB, a threshold effect was apparent. Dropping a limited amount of data had little impact, but dropping 75% of the data showed a significant decrease in the models’ predictive power.

Censoring the prediction period was performed to assess if short-term predictions are more accurate to make than long-term ones (Fig. 2b). Notably, censoring to 20-, 15-, or only 10-y prediction periods had a limited impact on model outcome.

### Impact of Validation Schemes

A range of analyses were performed where different training and test data sets were applied. Results for the base case model (the whole 2-cohort data set was split into a training and a test data set; internal validation) have been introduced already (Table 3). As discussed, the “apparent” performance measured in sample was always higher than the out-of-sample performance.

We further performed cross-center testing, where training was performed in one cohort and testing in the other. RPA was particularly sensitive to such cross-center testing: accuracy dropped to 0.62 when trained in Greifswald and tested in Kiel, but also more advanced models lost accuracy. The scenario of training in Kiel and testing in Greifswald was more accurate (XGB reached an AUC of 0.82 in this case). This asymmetry relates to the higher prevalence of tooth loss in Greifswald (0.14 ± 0.58) as compared with Kiel (0.11 ± 0.15).

Cross-temporal training and testing found AUCs close to the base case, indicating this as a possibly valid validation scheme. No model showed higher accuracy than the NIR regardless of the validation scheme.

## Discussion

The application of prediction models in medicine is supposed to increase health benefits, lower side effects, improve efficiency, and allow optimal resource allocation by estimating the chances of a positive or negative event a priori. To fulfill this purpose, prediction models should be robustly developed, reliable, and generalizable. In the present study, we demonstrated, using data from 2 cohorts, how various development and validation strategies for tooth loss prediction models affected model performance.

Various performance metrics were used to describe the predictive power of each model. It was apparent that the in-sample AUC, yielded from resampling and evaluating the developed model within the training data set, was high for all models and outperformed the out-of-sample AUC yielded in the test data set. This effect was evident for more complex models, such as RFO and XGB, and less obvious for simpler models, such as RPA and logR. Only reporting and displaying the in-sample model performance metrics seems insufficient to assess model performance, as it does not permit one to assess generalizability.

Also, the classification accuracy was apparently very high (often exceeding 0.90). However, none of the models significantly outperformed the NIR, which is the proportion of the data with the majority class. Randomly guessing the majority class (a tooth is retained) based on a priori knowledge (tooth loss is a rare event) was as accurate as complex prediction models. Our finding demonstrated the value of not only assessing accuracy values but also comparing them critically against the accuracy of chance (the NIR) and considering further metrics, such as sensitivity and specificity. Providing performance metrics, such as positive or negative predictive values or the area under the precision-recall curve, may also be considered when dealing with imbalanced data sets. The precision-recall curve does not suffer from class imbalance but is not common in dentistry so far (Saito and Rehmsmeier 2015). Future developments in dental prediction modeling may want to focus on models that specifically address the problem of an imbalanced data set. Moreover, investigators should consider replacing the use of randomly selected data with outcome-based sampling schemes, such as case-control designs, to increase the frequency of tooth loss in these data, as this may allow for better use of the modeling strategies that we examined.

We used a range of models, expecting that more complex models yield higher accuracies than less complex ones. Unexpectedly, we found only limited differences among models, and only in specific analyses was the simpler RPA clearly inferior to models such as RFO and gradient boosting. This is related to the fact that training on highly imbalanced data sets (as discussed) causes the model to learn representations of the majority class (tooth is retained) much better than those of the minority class (tooth is lost). Several methods to deal with class imbalance for machine learning have been proposed, such as over- and undersampling or synthetic minority oversampling (Sun et al. 2009). Applying such techniques was beyond the scope of this study.

Shrinkage of the data set was performed by dropping random patients from it. We found that data shrinkage affected model performance. Training on fewer events was found to severely affect the performance of more complex models (Steyerberg et al. 2017). We also expected that making predictions over longer periods is more difficult than over the short or midterm, as predictors may change (e.g., smoking status or PPD) either by behavior change or via treatment provided, for example. Hence, we tested how censoring affected model performance. However, the effect of prediction time was limited, possibly as baseline risks remained relatively stable long-term.

We also evaluated the impact of different validation schemes. As discussed, most studies in dentistry use some form of naive or internal validation (Faggion et al. 2007; Avila et al. 2009; Saminsky et al. 2015; Martinez-Canut et al. 2018; Schwendicke et al. 2018). We demonstrated that this may result in overfitting (especially for more complex models) and lead to high apparent (in-sample) performance. When models were externally validated across centers, model performance decreased (Zhan et al. 2014). Our findings demonstrate the need for careful external testing and awareness of statistical differences in the different populations to find out the true transportability (generalizability) of tooth loss prediction models. Notably, temporal validation, with training of models in 1 period and validation against another, seemed valid. We found the accuracies yielded by such cross-time validation to be rather stable. This was notable, as over time, secular (external) factors may apply on oral health (and risk of tooth loss), and treatment concepts may have changed to some degree (e.g., by technological advances). However, we want to highlight that this validation was possible within only the Kiel cohort, as here, data were gathered over >15 y.

This study has a number of strengths and limitations. First, we built on 2 large long-term followed cohorts of patients treated for periodontitis. As the cohorts were rather different in size, follow-up periods, and risk profiles, we assume the performed cross-center validation to be useful to assess transportability. In both cohorts, a similar treatment strategy was performed; extraction was executed very restrictedly during APT; and teeth doomed for extraction according to conventional textbook knowledge were retained. Thus, a number of teeth with a questionable or hopeless periodontal prognosis could be followed during SPT and were expected to be lost during maintenance (Graetz et al. 2011). A range of patient- and tooth-level predictors was employed, while admittedly, further parameters (even those beyond periodontal health), such as bruxism, diabetes, restorative status, and splinting of teeth, may be relevant for tooth loss (Martinez-Canut 2015; Graetz et al. 2018; Martinez-Canut et al. 2018). Our outcome, tooth loss, is relevant for patients from a quality-of-life perspective but also health economically. Second, a number of analyses were performed to assess the impact of modeling. To our knowledge, this is the first study in dentistry with this scope. Third, and as a limitation, the cohorts demonstrated high selection bias—for example, these subjects were very compliant (Lee et al. 2015) and smokers were underrepresented (Buchwald et al. 2013). Fourth, a rather conservative treatment regimen was applied. Testing the models in a very different cohort (practice based; e.g., with more teeth being removed or patients being less compliant) probably affects model performance. Also, as discussed, tooth loss was not necessarily due to periodontitis, as described, and the employed predictors, which mainly assess periodontal conditions, may have not been fully suited to capture other factors affecting tooth loss risk. Last, we evaluated only a specific set of modeling aspects, while many more affect model bias, stability, and performance. These should be evaluated by future studies.

## Conclusion

Within the limitations of this study, more complex models did not have advantages over simpler ones to predict tooth loss. Patients’ age and tooth loss at baseline as well as PPDs showed high variable importance. Internal validation came with a high risk of overfitting and only “apparently” high model performance. Shrinking the data set, thereby simulating smaller sample sizes, decreased the models’ predictive power. Overall, the models predicted tooth loss in patients with periodontitis with moderate discrimination ability. However, class imbalance significantly affected model performance, and none of the developed models would be useful when applied clinically. During modeling, rigorous development and validation should be applied and consequently reported.

## Author Contributions

J. Krois, F. Schwendicke, contributed to conception, design, data acquisition, analysis, and interpretation, drafted and critically revised the manuscript; C. Graetz, B. Holtfreter, P. Brinkmann, T. Kocher, contributed to data acquisition and interpretation, critically revised the manuscript. All authors gave final approval and agree to be accountable for all aspects of the work.

## Supplemental Material

DS_10.1177_0022034519864889 – Supplemental material for Evaluating Modeling and Validation Strategies for Tooth Loss Click here for additional data file. Supplemental material, DS_10.1177_0022034519864889 for Evaluating Modeling and Validation Strategies for Tooth Loss by J. Krois, C. Graetz, B. Holtfreter, P. Brinkmann, T. Kocher and F. Schwendicke in Journal of Dental Research
