# Supplementary material for: Evaluating Modeling and Validation Strategies for Tooth Loss
Source: J Dent Res. 2019 Jul 30;98(10):1088–95. doi: 10.1177/0022034519864889 (PMC6710618; doi:10.1177/0022034519864889)
Supplement: DS_10.1177_0022034519864889 – Supplemental material for Evaluating Modeling and Validation Strategies for Tooth Loss [file DS_10.1177_0022034519864889.pdf]

Evaluating Modeling and Validation Strategies for Tooth Loss

J. Krois, C. Graetz, B. Holtfreter, P. Brinkmann, T. Kocher, and F. Schwendicke

Appendix

Active and supportive periodontal therapy

Patients needed to have completed APT (T0-T1) and received SPT (T1-T2) for ≥ 9 years (Kiel) and ≥ 4 years (Greifswald), with minimum 1 visit/year including an annual documentation of probing-pocket depths (PPD) as well as a radiographic documentation at T0 and at the last documented visit of SPT (T2). All patients gave their informed consent for the analysis of their data documented during periodontal therapy. The study was approved by both local ethics committees (Kiel: D489/13; Greifswald: BB91/10).

Details on APT and SPT in the Kiel cohort have been described in detail elsewhere (Graetz et al. 2011; Graetz et al. 2017; Schwendicke et al. 2018). The treatment concept in APT in Greifswald was analogue to KI, using mainly non-surgical mechanical root debridement. Some differences regarding the adjunctive use of antibiotics and the indication of periodontal surgery applied; surgical treatment was only performed if the plaque index ≤ 30%, the age ≤ 75 years, the patient a non-smoker and no serious medical conditions present. In both centres, further treatments, e.g. endodontic treatment, splinting of mobile teeth, tunnelling procedures, molar root resections were carried out in individual cases. No pocket elimination surgery or osseous resection was undertaken.

SPT followed individualized intervals of three to twelve months, and included re-instruction/re-motivation of patients, individual oral hygiene, a professional tooth cleaning with scaling and root planing (SRP) of residual pockets and polishing by a dental auxiliary. If necessary, further treatments like open flap debridement with or without subsequent systemic antibiotic therapy were performed. In contrast to Kiel, each pocket in the Greifswald cohort with PPD ≥ 5 mm was re-instrumented independently of BOP. Also, for patients with severe periodontitis four visits per year were recommended.

Prediction models

Logistic regression is a well-established statistical method for modelling a binary outcome variable. The model belongs to the class of Generalized Linear Models (Nelder and Wedderburn

1972) and applies the logit link function to model a binary outcome as a linear combination of predictor variables.

The recursive partitioning algorithm (RPA) builds a binary decision tree model by a two-stage procedure (Therneau and Atkinson 1997). First the single predictor variable is found which best - we used the Gini impurity - splits the data into two groups. Then the data is separated and the first step is applied separately to each sub-group, recursively, until no improvement with respect to the Gini impurity can be made. In most applications the resulting full binary tree is very deep and tends to overfit the training data. Hence, in a second stage, cross-validation is applied to trim back the full tree.

Random forest (RFO) belongs to a class of ensemble models, where multiple models are combined to solve the same problem (Breiman 2001). The RFO algorithm basically builds a large collection of de-correlated decision trees. For construction of the collection of trees, RFO combines a bootstrap sampling approach (bagging) and a random selection of features. Each tree within the collections gives a prediction for an unseen data point. For a regression task, these predictions are averaged, and for a classification task, each tree casts a vote and the majority vote is used for predictions. RFO is in general robust to noise and overfitting. (Fawagreh et al. 2014).

Extreme gradient boosting (XGB) belongs, like RFO, to a class of tree-based ensemble models. Gradient boosting is a versatile algorithm, which is applied for classification, regression and ranking tasks. For classification, boosting combines the outputs of many weak classifiers to build up an ensemble of classifiers. Although a weak classifier is only slightly better than random guessing, by applying the weak classification algorithm to repeatedly modified versions of the data, a powerful ensemble of weak classifiers evolves over time (Hastie et al. 2009). The idea is to improve the model in a gradual, additive and sequential manner, so that subsequent classifiers are trained to classify observations that are not well classified by the previous classifiers. The term “gradient” was coined by Friedmann (2001) as the loss of the model is updated by applying gradient descent (Friedman 2001). XGB is a software implementation of gradient boosted decision trees designed for speed and performance (Chen and Guestrin 2016).

Sensitivity analysis

The data is analyzed on tooth level hence, we do not account for the within-mouth correlation of teeth from the same individual. Therefore we conducted a sensitivity analysis in which we

compared the predictive performance of a mixed effects logistic regression model and a standard logistic regression. We trained both models on the full data set of the baseline scenario (11651 teeth, 619 patients) and evaluated their performance (classification accuracy) via 10-fold cross validation. We used a t-test to test for a difference in the mean classification accuracy between the different models. Thereafter, the mixed effects logistic regression model is fitted to the full data set and corresponding coefficients are computed. We used the R library glm to fit a logistic regression and the R package lm4 to estimate a mixed effects logistic regression model with the patient ID as a random intercept.

The accuracies for each fold for each model are given in Table S1. The mean accuracy over 10 folds are 0.96 and 0.96 for the logistic regression and the mixed effects logistic regression model, respectively. The t-test does not yield a statistically significant difference in the accuracies (p=0.97).

Table S1. Results of the 10-fold cross validation for the standard logistic regression model and the mixed effects logistic regression model. A t-test yields no statistically significant difference in the accuracies (p=0.97).

	**Fold 1**	**Fold 2**	**Fold 3**	**Fold 4**	**Fold 5**	**Fold 6**	**Fold 7**	**Fold 8**	**Fold 9**	**Fold 10**	**mean**
**logistic regression**	0.96	0.95	0.96	0.97	0.96	0.92	0.96	0.98	0.96	0.94	0.96
**mixed effects logistic regression**	0.96	0.94	0.96	0.97	0.96	0.92	0.95	0.98	0.97	0.94	0.96

The model coefficients for the mixed effects logistic regression model are given in Table S2.

Table S2. Results from the mixed effects logistic regression model

**Random effects**	
patient-ID	1.648
**Coefficients**	**Log (Odds Ratio), (Standard error)**
Sex: male	0.104 (0.182)
Age at T1	0.017* (0.009)
Smoking status: former smoker	-0.209 (0.213)
Smoking status: smoker	0.321 (0.265)
Dental arch: upper	-0.682*** (0.091)
PPD at T1	0.418*** (0.036)
Bone loss: 25-50%	0.398*** (0.142)
Bone loss: 50-70%	1.238*** (0.158)
Bone loss: >70%	2.116*** (0.209)
Furcation involvement: Grade 2-3	0.448*** (0.149)
Mobility: 1	0.645*** (0.168)
Mobility: 2	0.966*** (0.248)
Mobility: 3	1.821*** (0.387)
Number of teeth	-0.084*** (0.022)
Tooth type: molar	-0.944*** (0.112)
Constant	-3.754*** (0.818)
Note:	*p<0.1; **p<0.05; ***p<0.01

References for the Appendix

Breiman L. 2001. Random forests. Machine learning. 45(1):5-32.

Chen T, Guestrin C. 2016. Xgboost: A scalable tree boosting system. Paper presented at: Proceedings of the 22nd ACM SIGKDD International Conference on Knowledge Discovery and Data Mining. ACM; San Francisco, California, USA.

Fawagreh K, Gaber MM, Elyan E. 2014. Random forests: From early developments to recent advancements. Systems Science & Control Engineering. 2(1):602-609.

Friedman JH. 2001. Greedy function approximation: A gradient boosting machine. The Annals of Statistics. 29(5):1189-1232.

Hastie T, Tibshirani R, Friedman J. 2009. The elements of statistical learning: Data mining, inference, and prediction. New York: Springer.

Nelder JA, Wedderburn RWM. 1972. Generalized linear models. Journal of the Royal Statistical Society Series A (General). 135(3):370-384.

Therneau TM, Atkinson EJ. An introduction to recursive partitioning using the rpart routines. 1997. https://cran.r-project.org/web/packages/rpart/vignettes/longintro.pdf; [accessed].
